# Radiopharmaceuticals for Persistent or Recurrent Uterine Cervix Cancer

**DOI:** 10.3389/fonc.2019.00560

**Published:** 2019-06-26

**Authors:** Charles A. Kunos, Jacek Capala, Elise C. Kohn, Susan Percy Ivy

**Affiliations:** ^1^Cancer Therapy Evaluation Program, National Cancer Institute, Bethesda, MD, United States; ^2^Radiation Research Program, National Cancer Institute, Bethesda, MD, United States

**Keywords:** radiopharmaceutical, targeted radioisotope therapy, uterine cervix cancer, cervical cancer, National Cancer Institute (NCI)

## Abstract

Uterine cervix cancers pose therapeutic challenges because of an overactive ribonucleotide reductase, which provides on-demand deoxyribonucleotides for DNA replication or for a DNA damage repair response. Ribonucleotide reductase overactivity bestows cancer cell resistance to the effects of radiotherapy and chemotherapy used to treat disease; but nevertheless, this same biologic overexpression provides opportune vulnerabilities relatively specific to uterine cervix cancers for new therapeutic strategies to take advantage. The discovery of human epidermal growth factor receptor 2 (*ErbB2* or HER2) overexpression on metastatic uterine cervix cancer cells provides an opportunity for clinical trials of targeted radiopharmaceuticals in combination with DNA damage response modifying drugs. The National Cancer Institute's clinical trial infrastructure and its experimental therapeutics portfolio can now offer clinical trial evaluation of molecularly-targeted and tolerated radiopharmaceutical-drug combinations for women with persistent or recurrent metastatic uterine cervix cancer. This article discusses the current thinking of the National Cancer Institute in regard to attractive radiopharmaceutical strategies for this disease and others.

## Introduction

Uterine cervix cancers afflict an estimated 13,170 American women and nearly 569,850 women worldwide (1, 2). It continues to be the fourth ranked cause of cancer-related death in women (2). This ranking persists because 36 percent of women have regional disease spread at initial diagnosis (International Federation of Gynecology and Obstetrics [FIGO] stage IB to IVA) and another 15 percent present first with distant disease (FIGO IVB) (3). A 5-years relative survival estimate for women with regional disease is 56 percent; it is only 17 percent for those with distant disease (3). New trial-tested treatments are needed for women advanced-stage regional and distant uterine cervix cancer.

The National Cancer Institute (NCI) has supported an early phase clinical trial infrastructure with a phase I trial emphasis for nearly 30 years (4). This program now goes by the moniker of the NCI Experimental Therapeutics Clinical Trials Network (ETCTN). Its clinical trial activities have involved more than 40 institutions and 300 investigators enrolling 33,485 patients over its 30-years history, accounting for eight percent of the nation's yearly clinical trial accrual (4). The ETCTN has as one of its goals the enrollment of a diverse research subject population into its early phase clinical trials for the sake of generalizability of treatment safety and efficacy. As has been observed in NCI's late phase networks (5), racial and ethnic minorities, elderly women, and those with socioeconomic barriers to care are underrepresented in ETCTN trials evaluating novel therapies for uterine cervix cancer (4). In the advent of leveraged partnerships between NCI's Cancer Therapy Evaluation Program and Radiation Research Program, there are at present opportunities for novel radiopharmaceutical phase I and II monotherapy or combination trials for women initially diagnosed with advanced-stage distant uterine cervix cancer.

Thus, this article offers perspective on new uterine cervix cancer radiobiology and relevant radiochemistry that have brought forward a prospect for radiopharmaceutical early phase clinical trials. It also provides an overview of pertinent uterine cervix cancer cell ribonucleotide reductase overexpression and its role in DNA replication and repair stress responses. The article discusses molecular approaches to targeted radiopharmaceutical delivery first in the promising molecular target of human epidermal growth factor receptor 2 (*ErbB2* or HER2) overexpression, and then, in combination with DNA damage response modifying drugs. Last, it offers perspective on the NCI community outreach efforts that underlie future ETCTN early phase clinical trials evaluating treatments for uterine cervix cancer in women of racial or ethnic minority or those with socioeconomic barriers to cancer care.

## Challenges and Opportunities

### Ribonucleotide Reductase Radiobiology

A balanced supply of deoxyribonucleoside diphosphates (dNDPs) is needed in mammalian cells for DNA replication and repair (6), and, this hallmark of cancer biology has the potential to be exploited by cancer therapies (7). A key molecular stakeholder for balanced supply of dNDPs is ribonucleotide reductase (RNR).

RNR activates in the S-phase of the cell cycle for DNA replication, or, after DNA base or single-strand or double-strand damage detection (6). RNR substitutes a hydroxyl for hydrogen in ribonucleoside diphosphates, generating narrowly-restricted quantities of equivalent dNDPs. RNR's large subunit α (M1) contains: (1) a catalytic pocket; (2) a dNDP-determining specificity pocket; and (3) a regulatory feedback-controlled activity pocket (7). RNR's M1 is found in all tumor cell cycle phases (6). RNR's M1 pockets can be drugged. Gemcitabine targets RNR's catalytic pocket (8); 5-fluorouracil (5-FU) disrupts biologic feedback to RNR's activity pocket (9). RNR's small subunit β (M2 or M2b) shuttles a vital tyrosyl radical to its catalytic pocket via proton-coupled electron transfer (10). Its M2 subunit is detected only in S-G2-M phases of the cell cycle, as it has a lysine-glutamate (KEN-box) amino acid motif that facilitates degradation by anaphase-promoting complex ligases in late mitosis (11). Its alternative p53-dependent small subunit, M2b, lacks a KEN-box sequence and therefore can be found in all cell cycle phases (12). Hydroxyurea and triapine inactivate RNR's tyrosyl radicals (13, 14). Early preclinical work of RNR inhibitors showed that uterine cervix cancer cells had a 17-fold rise in M2 expression about 18 h after irradiation and a 4-fold increase in dNDP output about 24 h later (15). Subsequently, it was discovered that RNR inhibitors arrest uterine cervix cancer cells at a G1-S-phase cell cycle restriction checkpoint for up to 18 h, impairs DNA damage repair for at least 6 h, and profoundly sensitized cancers to radiation–cisplatin cytotoxicity (16–18). High levels of RNR subunit expression suppress radiochemotherapy treatment response (19–21). In first-line clinical studies, approaches against advanced-stage regional uterine cervix cancer have found disrupting RNR overactivity during radiation-cisplatin exposure to be most beneficial (22–26). Molecular characterization came later.

### Targetable Mutation Biology

Given the central role of RNR in uterine cervix cancer, one might wonder whether pharmacogenomic targets (i.e., targetable mutations in putative oncogenes) represent a good source of anticancer drug targets at all in this disease. There are at least two pharmacogenomic aspects that are different in uterine cervix cancer cells compared with normal cells, which in turn introduces attractive drug targets that can (and indeed currently are) being exploited for new uterine cervix cancer treatments.

Uterine cervix cancer molecular characterization approaches through 2019 have focused primarily on single-gene mutations in cell cycle proliferation pathway genes (predominantly *PIK3CA*—E542K or E545K [26% of 192 sampled]) (27). Novel recurrent focal amplification events in the human epidermal growth factor receptor 2 (*ErbB2* or HER2) at chromosome 17q12 have also been detected in uterine cervix cancers (17%) (27). Single-gene mutations or gene amplifications have multiple roles in the promotion of cancer cell growth by being driver of proliferation mutations and by being evaders of cell apoptosis. The effects of specific single-gene mutations on a cell's fate are still not well-understood. Functional single-gene or gene amplification biomarkers promoting RNR overactivity are active areas of research. Better definition of these biomarkers in a uterine cancer cell-specific or mutation-specific manner might inform the evaluation of radiopharmaceutical-drug combination trials.

### Impact of Uterine Cervix Cancer Disease Presentation on Radiopharmaceutical Clinical Development

The uterine cervix, which is the lowermost anatomical portion of the uterus, forms a cylindrical-shaped organ made up of epithelium and stroma. In 2018, the International Federation of Gynecology and Obstetrics (FIGO) updated the 2014 uterine cervix cancer staging system for resource-permitting clinical and imaging assessments to determine initial cancer stage (28, 29). Now ultrasound, computed tomography, magnetic resonance imaging, or positron emission tomography furnish supportive information on tumor size, lymph node disease status, and regional or distant spread. Two striking changes were made (Table 1)—disease confined to the uterine cervix (stage IB) now has three incremental tumor size categories, and, a new category (IIIC) subdivided into disease detected in pelvic lymph nodes (IIIC1) or found in para-aortic lymph nodes (IIIC2). Uterine cervix cancer patient population statistics have validated survival differences among these categories (30).

**Table 1:** New 2018 uterine cervix cancer staging system.

**Stage**	**2014 staging system**	**2018 staging system**
1B1	Clinical tumors no > 4 cm in size	Clinical tumors no > 2 cm in size
1B2	Clinical tumors > 4 cm in size	Clinical tumors 2 cm or more in size and no > 4 cm in size
1B3	Not coded	Clinical tumors 4 cm or more in size
IIIC1	Not coded	Clinical pelvic lymph node metastasis only^*^
IIIC2	Not coded	Clinical para-aortic lymph node metastasis^*^

* *Detected by radiographic (r) or histopathologic (h) means. Adapted from reference (29)*.

Uterine cervix cancers are relatively radioresistant [Table 2, (31–33)], requiring 7,500–8,000 centigray or more radiation dose to overcome RNR (19–21). Early clinical reports for this disease before knowledge of RNR overactivity suggested that hypoxic, bulky uterine cervix cancer tumors were better treated by extrafascial hysterectomy than by intracavitary brachytherapy (34, 35). The notion that cancer cells invaded uterine cervix stroma 10 millimeters or more away from visible lesions strengthened the extrafascial hysterectomy approach [Table 3, (36)] and adds perspective for the “reach” of targeted radiopharmaceuticals tagging and killing occult microscopic spread of disease away from the primary tumor. Two uterine cervix cancer trials offer outcome data, where surgery tackled occult locoregional disease, to inform next generation radiopharmaceutical trials in this disease.

**Table 2:** Surgicopathological uterine cervix cancer clinical trials.

**Study**	**Phase**	**Disease setting (*n*)**	**Treatment arm**	**Most common acute grade ≥3 AEs**	**Efficacy**	**Reason for notability**
GOG-049 (36)	None	1st-line stage I (>3 mm) uterine cervix cancer (645)	Radical hysterectomy, then adjuvant physician choice	Not reported	R0: 97%	Identified independent risk factors for nodal metastasis
MILAN (33)	III	1st-line stage IB or IIA uterine cervix cancer (170)	Radical hysterectomy, then adjuvant physician choice	Any grade 2–3 (19%)	R0: 89%	Randomized hysterectomy (±irradiation) vs. irradiation
GOG-071 (31)	III	1st-line stage IB uterine cervix cancer (131)	EBRT + BRACHY, then extrafascial hysterectomy	Gastrointestinal (6%) or Genitourinary (3%)	pCR: 48%	Randomized hysterectomy after irradiation
GOG-123 (32)	III	1st-line stage IB (>4 cm) uterine cervix cancer (186)	EBRT + BRACHY, then extrafascial hysterectomy	Gastrointestinal (5%) or Genitourinary (3%)	pCR: 41%	Randomized neoadjuvant irradiation prior to hysterectomy
GOG-123 (32)	III	1st-line stage IB (>4 cm) uterine cervix cancer (183)	EBRT + BRACHY + cisplatin days 1, 8, 15, 22, 29, 36, then extrafascial hysterectomy	Gastrointestinal (14%) or Genitourinary (2%)	pCR: 52%	Randomized neoadjuvant radiochemotherapy prior to hysterectomy
CLEVELAND (19)	None	1st-line advanced-stage uterine cervix cancer (51)	EBRT + BRACHY + cisplatin days 1, 8, 15, 22, 29, 36 then post-therapy cervical sampling	Not reported	pCR: 84% PET CR: 76%	Linked post-therapy PET and post-therapy treatment response

*AEs, adverse events; BRACHY, intracavitary brachytherapy; EBRT, external beam radiotherapy; GOG, Gynecologic Oncology Group; pCR, surgicopathological complete response rate (absence of any uterine cervix tumor); PET CR, ^18^F-flurodeoxyglucose positron emission tomography complete response rate; R0, hysterectomy with disease-free surgical margins*.

**Table 3:** Uterine cervix cancer microscopic invasion^*^.

**Depth**	**Cases**	**Proportion (%)**
Microscopic extent no > 5 mm	177	28
Microscopic extent > 5 mm and no > 10 mm	238	38
Microscopic extent > 10 mm and no > 15 mm	135	21
Microscopic extent > 15 mm and no > 20 mm	49	8
Microscopic extent > 20 mm	31	5

* *NCI Gynecologic Oncology Group protocol #049 (n = 630) (36)*.

Between 1984 and 1991, 256 women enrolled on a randomized trial in women with stage IB uterine cervix cancers (31). 254 [99%] of 256 received external beam radiotherapy and intracavitary brachytherapy. 123 (93%) of 132 women underwent a randomly-allocated post-radiation extrafascial hysterectomy. The post-radiation pathological complete response rate (pCR) in those undergoing extrafascial hysterectomy was 48 percent (31). The cumulative incidence of a regional relapse was 14 percent after radiotherapy-hysterectomy and 27 percent after radiotherapy alone. Six years overall survival estimates were 62 percent after radiotherapy-hysterectomy and 60 percent after radiotherapy alone, achieving a non-significant hazard ratio of 0.84 (31).

Between 1992 and 1997, another clinical trial randomized 369 women with bulky stage IB uterine cervix cancers four centimeters or larger to receive either external beam radiotherapy and intracavitary brachytherapy alone (*n* = 186) or the same radiotherapy plus weekly cisplatin (40 mg/m^2^) (*n* = 183) (32). Long-term outcome data have been updated (37). Compliance with radiotherapy was high (366 [99%] of 369) (32). Extrafascial hysterectomy was done in 168 (90%) radiotherapy-only patients and in 175 (96%) radiotherapy-cisplatin patients (32). The pCR rate after radiotherapy-hysterectomy was 41 percent and after radiotherapy-cisplatin-hysterectomy was 52 percent (37). The cumulative incidence of a regional relapse was 11 percent after radiotherapy-cisplatin-hysterectomy and 24 percent after radiotherapy-hysterectomy [statistic not reported; (37)]. Six years overall survival estimates were 78 percent after radiotherapy-cisplatin-hysterectomy and 64 percent after radiotherapy-hysterectomy, achieving a significant hazard ratio of 0.63 [*P* < 0.015; (37)].

The NCI recognizes that treatment approaches to regional or distant uterine cervix cancer disease can be diverse, involving surgery, radiotherapy, or chemotherapy alone or in combination. When considering clinical development of radiopharmaceuticals for regional or distant stages of this disease, lessons learned from prior surgicopathological studies inform which disease settings make rational sense. Intracavitary brachytherapy like that practiced in clinical trials might deliver meaningful sterilizing radiotherapy dose one centimeter away or closer to the intracavitary tumor implant. Targeted radiopharmaceuticals might be capable of extending that radiotherapy dose “reach.” Certainly, well-targeted radiopharmaceuticals could affect regional and elsewhere distant uterine cervix cancer disease beyond dose applied by a brachytherapy applicator. The NCI offers the perspective that advanced-staged regional disease might benefit from external beam radiochemotherapy and intracavitary brachytherapy followed by targeted radiopharmaceuticals, as might be in line with pending findings of the OUTBACK chemotherapy trial (clinicatrials.gov, NCT01414608). For advanced-stage distant disease, monotherapy or combination therapy clinical trials of targeted radiopharmaceuticals are attractive because of the well-characterized responses of uterine cervix cancer cells to irradiation. Nuances of these two approaches are explained in the next section of the article.

## Perspectives on Radiopharmaceuticals for Uterine Cervix Cancer

### NCI Infrastructure

Cancer clinical trials offer essential research to find better means to prevent, treat, control, and cure cancer. NCI's strategy to grow its targeted radiopharmaceutical clinical trial portfolio involves new methods for scientific evaluation, medical supervision, and other infrastructure needs necessary for early or late phase development (38, 39). Value-added tactics, such as empowering nurse navigators to assist in uterine cervix cancer trial accrual (40), need more thought before executing radiopharmaceutical trials in this disease. Leveraged programmatic collaboration, cost-sharing plans amongst its partners, radiopharmaceutical drug product formulation and distribution logistics, and a full understanding of how radiopharmaceutical treatment plans are implemented by rostered authorized users (radiation oncologists or nuclear medicine physicians) are well-known preconditions for successful trial implementation. From the NCI's perspective, effort is expended on a targeted radiopharmaceutical clinical development program because it provides efficient, safe, and cost-effective study of these types of new experimental agents. Implementing radiopharmaceutical early phase trials in the ETCTN is a first step.

Take for example the current ETCTN-sponsored community oncology outreach in Appalachia. Here, two ETCTN lead sites plan enrolling women newly diagnosed with advanced stage uterine cervix or vaginal cancer to a phase I trial of oral triapine plus radiation-cisplatin in the first-line patient setting (clinicaltrials.gov, NCT02595879). Consider that Appalachia consists of 410 counties in 13 states encompassing 22 million Americans, or about eight percent of the total American population (41). Death from uterine cervix cancer disease occurs within 1 year in about four (36%) of every 11 Appalachian women diagnosed, outpacing mortality most elsewhere in the United States (42). Understanding better recruitment patterns, perceived barriers to trial accrual, and logistical challenges like patient transportation needs, phase I infusion center space, or frequency of blood or tissue acquisition in Appalachia will serve the effort to bring forth new treatments to women in the minority or underserved population. NCI plans study of unique opportunities that rise up for radiopharmaceuticals targeting uterine cervix cancer in its ETCTN, and so, an effort to bring these types of trials to Appalachia has begun.

### Targeted Radiopharmaceuticals and Potential Radiosensitizing Drug Combinations

**Figure 1:**
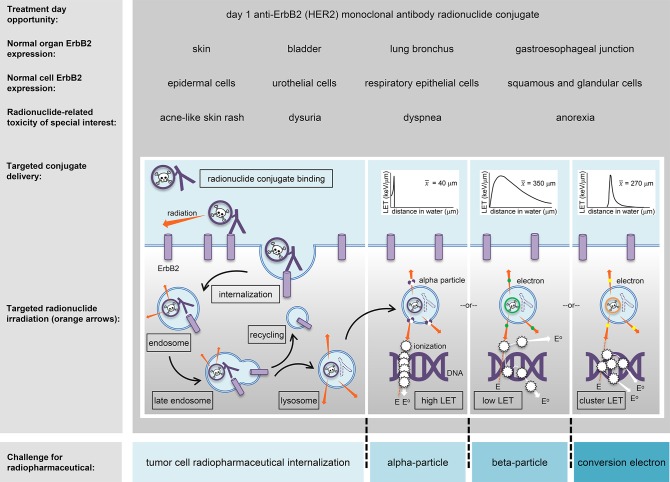
An HER2 (*ErbB2*)-targeted radiopharmaceutical is charted in relation to thorium-227 radionuclide delivery and adverse events (toxicities) of special interest. The skin, bladder, lung bronchus, and stomach-esophagus show normal molecular expression of HER2 and are listed together with specific cell subtypes that might have detectable levels of the receptor for off-target radiopharmaceutical localization. Marked in boxes are the steps in antibody conjugate processing likely engaged in intended irradiation of uterine cervix cancer cells or in unintended toxicity of normal cells. Challenges for a radiopharmaceutical and its radiobiology (like particle range [inserts]) are illustrated for alpha-particles, beta-particles, and conversion electrons (blue boxes). E, energy track path; E°, energy track path after ionization; *ErbB2*/HER2, human epidermal growth factor receptor 2; keV, kiloelectron volt; LET, linear energy transfer; x-bar, average.

Radiotherapy can be given by treatment beams external to the body, by applicators internal to a tumor or the body, or by ingested or intravenous radiopharmaceuticals targeting tumors via the body's bloodstream. Targeted radiopharmaceuticals aim the transfer of energy-rich alpha-particles (helium nuclei), beta-particles (electrons), or conversion electrons, to cancer cells residing in soft tissue or in bone-occupying tumors (Figure 1). For the purpose of this article, targeted transfer means that a ligand, such as a peptide or an antibody, traps a radiopharmaceutical for a short duration during its decay with great affinity onto cancer cells more than normal cells. Alpha-particles, beta-particles, and conversion electrons transfer energy in characteristic ways, a process coined linear energy transfer or LET (Figure 1 insets). LET describes the ionizing power of radionuclides to donate “damaging” excitation energy to cells traversed per unit distance (or, depth of tissue penetration) (43). Alpha-particle radiopharmaceuticals like radium-223 or thorium-227 deposit highly ionizing energy in a linear track between 40 and 130 μm, or about 1–10 tumor cell diameters. Beta-particle emitters like lutetium-177 deposit lower ionizing energy in divergent tracks over 350 μm or nearly 27 tumor cell diameters. Internal conversion electrons emitted from radionuclides, but not from its nucleus, deposit ionizing energy in clusters at specific intervals, like tin-117 m which clusters at 270 μm or 21 tumor cell diameters. So far, five alpha-particle emitting conjugated radiopharmaceutical have been recruited to NCI's experimental therapeutics portfolio (ctep.cancer.gov)—including the anti-HER2 monoclonal antibody trastuzumab-thorium-227 conjugate (44).

The human epidermal growth factor (HER) family, known also as the *ErbB* receptor tyrosine kinase family, comprises the epidermal growth factor receptor (EGFR or *ErbB1*), HER2 (or *ErbB2*), HER3 (or *ErbB3*), and HER4 (or *ErbB4*) cell surface receptors (45). Breast and gastric cancers overexpress HER2 (44). HER2 overexpression might be found in three percent of treatment-naïve uterine cervix cancers, but up to a high of 21 percent in recurrent cases (46–48). Dimerization of HER2 with other members of the ErbB family triggers the mitogen-activated protein kinases (MAPK) and phosphatidylinositol 3-kinase (PI3K)/protein kinase B (Akt) pathways to engage cell-fate decisions in proliferation, differentiation, and apoptosis (45). Overexpression associates with metastatic progression (46); therefore, anti-HER2 inhibitors have been developed as targeted anti-cancer drugs. Lapatinib, afatinib, and neratinib, all HER2 tyrosine kinase inhibitors, block functional signaling but are associated with poor clinical efficacy in a limited number of uterine cervix cancer patient-treated women [4 [5%] of 78 and 0 [0%] of 2, respectively (49, 50)]. The monoclonal anti-HER2 antibody trastuzumab alone demonstrates low monotherapy efficacy [1 [3%] of 35; (46)]. The anti-HER2 antibody-drug conjugate (ADC) trastuzumab emtansine, which delivers the microtubule poison mertansine (DM1) via the trastuzumab targeting antibody ligand, has not yet been administered to uterine cervix cancer patients.

An antibody–thorium-227 radionuclide conjugate has been created to target HER2-positive cancer cells using the humanized anti-HER2 IgG1 antibody trastuzumab (44). It has undergone preclinical xenograft mouse modeling and toxicology for localized and disseminated breast and gastric cancer (17). The trastuzumab-thorium-227 drug formulation is now positioned to enter the clinic for first-in-human pharmacokinetic and pharmacodynamic studies. Acne-like skin rash, anorexia, dyspnea, and dysuria are all toxicities encountered after anti-HER2 therapies for cancer (Figure 1) and might rise to adverse events of special interest that will require additional toxicity monitoring and reporting in NCI trials. NCI's radiopharmaceutical clinical development plan envisions antibody–thorium-227 radionuclide conjugate trials that test whether this agent can be safely combined with triapine (RNR inhibitor), nedisertib (M3814, DNA-protein kinase inhibitor), ceralasertib (AZD6738, ataxia-telangiectasia mutated and Rad3-related kinase (ATR) inhibitor), adavosertib (AZD1775, WEE1 inhibitor), telaglenastat (CB839, glutaminase-1 inhibitor), ivosidenib (AG120, isocitrate dehydrogenase 1 inhibitor), or enasidenib (isocitrate dehydrogenase 2 inhibitor).

### Navigating Radiopharmaceutical Clinical Development

**Figure 2:**
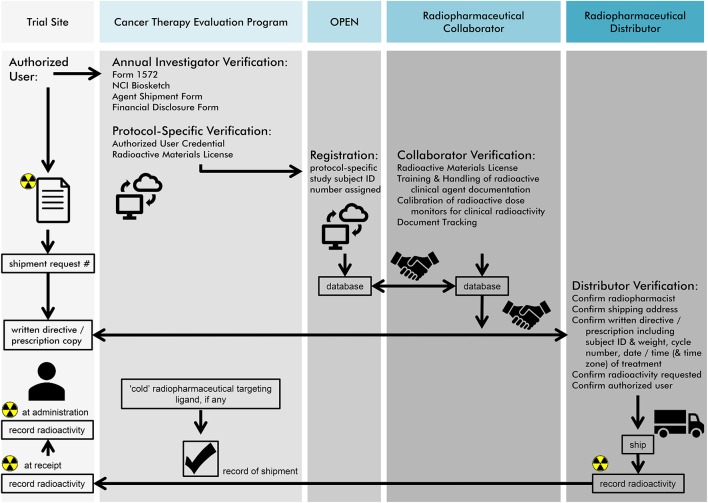
NCI envisions a radiopharmaceutical agent order process as charted in this figure for site users, its Cancer Therapy Evaluation Program (CTEP) pharmaceutical management branch (PMB), its oncology patient enrollment network (OPEN), and anticipated tasks for its radiopharmaceutical collaborators or distributors. First, authorized users must undergo an annual investigator and protocol-specific verification. Second, an OPEN-assigned study subject identification and a subject's weight are used to request radiopharmaceutical agent for a pre-planned date of treatment. Third, a “handshake” must occur between NCI, its radiopharmaceutical collaborators and distributors for agent order processing. If a non-radioactive (or “cold”) radiopharmaceutical targeting ligand is used, CTEP manages the shipment of this molecular entity to the site. Last, NCI recommends that three minimum data elements for radiopharmaceuticals must be recorded—(i) agent radioactivity at initial dose shipment, (ii) agent radioactivity at site receipt, and (iii) agent radioactivity upon administration to the patient. The radiopharmaceutical authorized user or designee must maintain an appropriate NCI Investigational Agent (Drug) Accountability Record (DARF) and separate NCI Investigational Agent Accountability Records for each radiopharmaceutical agent, strength, formulation, and ordering investigator.

The NCI recognizes that there are opportunities for radiation-agent dose, schedule, exposure, and effect study, and a leveraged cross-programmatic approach is desired. A network of preclinical laboratories has been considered to offer cell-based and high-throughput tissue microarray (TMA) technology for radiopharmaceutical target ligand validation. As compared to conventional histopathology studies where single tissue biospecimens are inspected independently, TMAs offer target ligand expression in greater sample quantity in a single analytic test. TMA workflow includes (i) tissue sample selection inclusive of positive and negative ligand expression controls, (ii) manufacture of the TMA block with tissue cores, (iii) quality control of sectioned blocks (i.e., ensuring presence of tumor in each core), (iv) immunohistochemical analyses of TMA sections, (v) photography, (vi) qualitative or quantitative scoring of TMA tissue cores/images for ligand expression, and (vii) statistical analyses (20, 21). Building upon this kind of preclinical data, the NCI Experimental Therapeutics program offers a one-stop entry gateway to radiopharmaceutical development (https://next.cancer.gov). After deliberation by NCI intramural clinical, translational, and basic radiobiology experts, the NCI requests Project Team Member Applications (PTMAs) from among interested extramural members of the ETCTN, the National Clinical Trials Network (NCTN), or others, in an effort to formulate an initial radiopharmaceutical drug development plan to be carried forward in clinical trials. As an example, the project team drafting radiopharmaceutical drug development plans for radium-223, included radiation and medical oncologists, nuclear medicine physicians, and radiation medicine physicists from NCI working group, resulting in proof-of-concept radium-223 and DNA damage response-modifying agent phase I and II trials relying on a sophisticated radiopharmacovigilance (Figure 2). Letters of Intent (LOIs) describing rationale for radiopharmaceutical plus agent dose-escalated phase I trials or randomized-arm radiopharmaceutical-agent phase II trials could be submitted for NCI and pharmaceutical collaborator approval and trial implementation (https://ctep.cancer.gov). LOIs with strong rationale and preclinical data that describe therapy that considers radiopharmaceutical-agent pharmacodynamics and real-day patient logistics are often met favorably in the approval process. Investigator, NCI, and pharmaceutical collaborator communication and expert critique before LOI submission often strengthen radiopharmaceutical-agent development plans, making approval more likely.

## Conclusion

In summary, this perspective article discusses the vision of radiopharmaceutical clinical development as related to use in women with persistent or recurrent uterine cervix cancer. It offers insight into approach alone or in combination with DNA damage response modifying drugs. It also offers perspective on NCI community outreach efforts in future ETCTN early phase radiopharmaceutical clinical trials, like in Appalachia, for women with persistent or recurrent uterine cervix cancer as a clinical trial demonstration project. The education of both patients and radiation oncologists or nuclear medicine physicians regarding the use of radiopharmaceuticals remains essential to the beneficial clinical development of these agents in women with uterine cervix cancers.

## Data Availability

The raw data supporting the conclusions of this manuscript will be made available by the authors, without undue reservation, to any qualified researcher.

## Ethics Statement

The research presented in this article involved the collection or study of existing data, documents, and records that were publicly available, or the information was recorded by NCI in such a manner that trial subjects cannot be identified directly or through identifiers linked to the subjects. The research is regarded exempt from Institutional Review Board oversight.

## Author Contributions

CK, JC, EK, and SI contributed to the collection and review of any data, analysis, authentication, and the writing and approval of this manuscript. The views expressed are those of the authors and not those of the U.S. Federal Government. Links or discussions of specific treatments do not constitute endorsement.

## Conflict of Interest Statement

The authors declare that the research was conducted in the absence of any commercial or financial relationships that could be construed as a potential conflict of interest.
